# Coagulation profile and platelet count in pediatric patients with extrahepatic portal vein obstruction

**DOI:** 10.1590/1984-0462/2025/43/2025123

**Published:** 2025-12-12

**Authors:** Gabriela Barbosa Unger, Roberta Vacari de Alcantara, Maria Angela Bellomo Brandão, Adriana Maria Alves De Tommaso, Roberto Massao Yamada, Gabriel Hessel

**Affiliations:** a Universidade Estadual de Campinas, Campinas, SP, Brazil.

**Keywords:** Portal vein, Thrombosis, Pediatrics, Blood coagulation, Veia porta, Trombose, Pediatria, Coagulação sanguínea.

## Abstract

**Objective:**

The aim of the study was to study coagulation abnormalities and hematimetric indices in pediatric patients with extrahepatic portal vein obstruction (EHPVO).

**Methods:**

This was a retrospective and longitudinal study, which included 93 patients diagnosed with EHPVO treated between January 1991 and December 2023 at a reference center for pediatric liver diseases. The research was carried out by collecting data from the medical records of these patients, who were divided into three groups: G1 (n=78; EHPVO without surgery), G2 (n=4; EHPVO with surgery without splenectomy), and G3 (n=11; EHPVO with splenectomy).

**Results:**

The mean age at admission was 5.48 years, with 64.5% male patients and an average follow-up time of 10.42 years. At admission, the prevalence of prolonged R (thromboplastin time Ratio) and INR (international normalized ratio) was 52.32% and 26.8%, respectively; anemia was observed in 67.7% of patients, and thrombocytopenia in 70.45%. In the comparison of each variable between admission and the last follow-up, it was observed that for R, INR, and platelets, there was no significant difference in group 1. However, there was a significant difference in group 3, with a reduction in R and INR at the last follow-up compared to admission, and a significant increase in platelets between admission and the last follow-up. Owing to the reduced sample size, the outcomes of group 2 were excluded from the between-group comparisons due to limitationsin statistical power.

**Conclusions:**

The percentage of anemia, thrombocytopenia, and prolonged INR at admission was high. Splenectomy resulted in an increase in platelet count and a reduction in R and INR. Therefore, the alteration in INR and R at admission is likely not due to the consumption of coagulation factors in the cavernoma region, but rather, probably in the spleen.

## RESUMO

**Objetivo:**

Estudar as anormalidades da coagulação e de índices hematimétricos em pacientes pediátricos com obstrução da veia porta extra-hepática (OVPEH).

**Métodos:**

Tratou-se de estudo retrospectivo e longitudinal, que incluiu 93 pacientes com diagnóstico de OEHVP atendidos entre janeiro de 1991 e dezembro de 2023 em um centro de referência para doenças hepáticas pediátricas. A pesquisa foi realizada por meio da coleta de dados dos prontuários desses pacientes, que foram divididos em três grupos: G1 (n=78; OVPEH sem cirurgia), G2 (n=4; OVPEH com cirurgia sem esplenectomia) e G3 (n=11; OVPEH com esplenectomia).

**Resultados:**

A média etária na admissão foi de 5,48 anos, sendo 64,5% do sexo masculino, com tempo médio de seguimento de 10,42 anos. Na admissão, a frequência de Razão Normalizada Internacional (RNI) e Razão de Tempo de Tromboplastina (R) alargados foi de 52,32 e 26,8%, respectivamente, de anemia 67,7% e de plaquetopenia 70,45%. Na comparação de cada variável em relação ao valores na admissão e na última consulta, observou-se que para R e RNI e plaquetas não houve diferença significativa no grupo 1, mas houve diferença significativa para o grupo 3, com redução de R e RNI na última consulta em relação à admissão e aumento significativo de plaquetas entre admissão e última consulta. Dado o tamanho reduzido da amostra, os desfechos do grupo 2 não foram incluídos nas comparações entre os grupos em razão da limitação do poder amostral

**Conclusões:**

Foi alto o percentual de anemia, plaquetopenia e alargamento de RNI na admissão. A esplenectomia refletiu em elevação na contagem de plaquetas e redução do R e RNI. Logo, a alteração de RNI e R na admissão não se deve ao consumo de fatores de coagulação na região do cavernoma, mas sim, provavelmente, no baço.

## INTRODUCTION

Extrahepatic portal vein obstruction (EHPVO) is a vascular disorder of the extrahepatic portal vein, which may or may not involve the intrahepatic portal veins, the splenic vein, and the superior mesenteric vein. Portal vein obstruction related to chronic liver disease or neoplasia is not classified as EHPVO.^1^

The pathophysiology of EHPVO is based on the formation of a thrombus that causes occlusion of the extrahepatic portal vein. As a result of this obstruction, there is an increase in portal pressure and the development of collateral vessels around the portal vein, which are observed on angiography as cavernoma- tous transformation.^2^ This mechanism of collateral vessel formation aims to maintain viable portal perfusion even with an obstructed portal vein. In general, EHPVO presents asymptomatically, and the time between thrombus formation and the development of collateral circulation is, on average, 3 weeks.^3^

In about 50% of patients, the cause of EHPVO is not identified. When the etiology is known, it can be divided into three categories: 1^st^ Conditions that lead to direct injury of the portal vein causing obstruction, such as omphalitis, umbilical vein catheterization, neonatal peritonitis, abdominal trauma, or surgery on the vein, cysts, and tumors; 2^nd^. rare developmental anomalies, such as portal vein stenosis, portal vein atresia, and agenesis; and 3^rd^ factors indirectly related to portal vein thrombosis, such as neonatal systemic sepsis of extra-abdominal origin, dehydration, multiple transfusions, hypercoagulable states, and myeloproliferative disorders.^4^ The most common identified cause is a history of umbilical catheterization, a condition that can lead to direct injury to the portal vein and thrombosis.^5^ EHPVO is an important cause of portal hypertension in pediatric patients, ranging from 20.3 to 63.0%.^6,7^

The diagnosis is confirmed by imaging tests such as Doppler ultrasound, computed tomography, and magnetic resonance imaging. Doppler ultrasound is the test of choice due to its high sensitivity (70”90%) and specificity (99%) for detecting portal cavernoma.^6,7,8^

Treatment varies depending on the clinical presentation. In the case of recent obstruction, anticoagulation is a viable choice. However, in most cases, it is a chronicobstruction, whose treatment includes control and prophylaxis of bleeding from esophagogastric varices and portal hypertension gastropathy. Surgical treatment with systemic porto shunt is indicated in the following situations: failure to control upper gastrointestinal bleeding, significant hypersplenism, and hepatopulmonary syndrome. There are some relative indications for surgery, including symptomatic splenomegaly, large-caliber varices with high bleeding risk, symptomatic portal cavernoma cholangiopathy, and failure to gain weight and height.^1^ Recently, some authors have recommended earlier surgery and opted for the Rex shunt procedure with the goal of restoring intrahepatic portal blood flow and preventing complications such as liver dysfunction and hepa- topulmonary syndrome.^9^

The emphasis in most publications has been on controlling upper gastrointestinal bleeding and the indications for surgical treatment.^10,11,12,13,14,15^ However, there are other complications described, such as hypersplenism and coagulation abnormalities, that have not been extensively studied in the pediatric age group. The first authorsto study the topic reported a 25% prevalence of prolonged international normalized ratio (INR) and explained this alteration by the consumption of coagulation factors at the site of the cavernoma that develops after the thrombotic event.^16^ There are few studies on coagulation abnormalities and hematimet- ric indices. One study concluded that there are no coagulation disorders in EHPVO, while three other studies found abnormalities in prothrombin time, partial thromboplastin time, and platelet function.^17,18,19,20^ The aim of this study was to investigate coagulation abnormalities and hematimetric indices in pediatric patients with EHPVO.

## METHOD

This is a cohort study with retrospective data analysis. The patients included in this study were treated at the pediatric hepatology outpatient clinic of the ClinicalHospital of the University of Campinas (UNICAMP), a tertiary referral center for liver diseases in the pediatric age group. A retrospective analysis of medical records was performed for 101 patients diagnosed with EHPVO who were admitted before the age of 18 during the period from 1991 to 2023. Patients who did not have coagulation and hematimetric test results at admission and at the last follow-up were excluded; a total of eight patients were excluded due to incomplete data. The patients who were admitted and met the inclusion criteria were divided into three groups: group 1 (EHPVO without surgery), group 2 (EHPVO with surgical shunt but without splenectomy), and group 3 (EHPVO with splenectomy associated or not with surgical shunt). The indication for surgery was based on criteria defined in the guidelines, with the first recommending surgery only for advanced cases, while the most recent one suggests surgical intervention during the early stage of the disease.^1,9^

Data collection was performed through a review of medical records. The data collection form included the following information: group (G1, G2, or G3), sex, age at admission to the outpatient clinic at UNICAMP, age at the last follow-up (up to December 2023), follow-up time, etiology of EHPVO, need for surgery, type of surgery, age at the time of surgery, results of R (thromboplastin time ratio; reference value (RV) <1.3), INR (international normalized ratio; RV <1.25), hemoglobin (RV≥12), platelets (RV≥150,000), and splenic index (RV <1; values obtained through ultrasound as the ratio between the longitudinal dimension of the spleen in cm and the normal longitudinal dimension of the spleen for that age group in cm) at admission and at the last follow-up until December 2023.^21^

To describe the sample profile according to the study variables, frequency tables of categorical variables were constructed with absolute values (N) and percentages (%). For continuous variables, descriptive measures (mean, standard deviation, median, and quartiles) were obtained. For comparison of proportions, the chi-square test or Fisher’;s exact test was used when necessary. To compare numerical measures between two groups, the Mann-Whitney test was applied, and for comparison among three groups, the Kruskal-Wallis test followed by Dunn’s test was used to locate the differences when necessary. To assess the relationship between numerical variables, Spearman’s rank correlation coefficient was applied. For comparison of numerical measures evaluated at two different time points within the same group, the Wilcoxon test for related samples was used. Repeated measures analysis of variance (ANOVA) with rank transformation was applied for the comparison of groups and time points for numerical variables. The significance level adopted for the study was 5%. This study was approved by the Research Ethics Committee of the University of Campinas (approval number 6,327,507), and informed consent or assent was obtained from all patients who were under clinical follow-up during the study period.

## RESULTS

The study included 93 patients, who were divided into the following groups: G1, n=78; G2, n=4; and G3, n=11. The predominant surgery in G2 was the portomesenteric shunt (Rex shunt), and the predominant surgery in G3 was the proximal splenorenal shunt with splenectomy. The mean age at admission was 5.48±4.77 years, with 60 male patients (64.5%), and the average follow-up time was 10.42±6.18 years. The identified risk factors were: umbilical catheterization in 58 out of 93 patients (62.4%), sepsis in 12 out of 93 (12.9%), and thrombophilia in 2 out of 93 (2.1%). An idiopathic etiology was considered in 21 out of 93 patients (22.6%). At admission, the frequency of enlarged INR and R was 52.32% and 26.8%, respectively; anemia was present in 67.7%, and thrombocytopenia in 70.45%. The demographic data of the sample and the hematimetric and coagulation test results are presented in Table 1.

**Table 1:** Demographic data, follow-up time in years, and results for hemoglobin, thromboplastin time ratio, international normalized ratio, platelets, and splenic index in groups 1, 2, and 3.

Variable	Group 1	Group 2	Group 3	p-value
Age at admission (years)				
N	78	4	11	0.1670^*^
Median (1st”3rd. quartiles)	5.25 (2.94”8.44)	3.88 (3.25”4.35)	2.92 (2.58”4.33)	
Age at last consultation (years)				
N	76	4	11	0.6453^*^
Median (1st”3rd. quartiles)	16.54 (13.60”19.67)	15.71 (14.48”16.56)	18.17 (13.46”22.37)	
Follow-up time (years)				
N	76	4	11	0.2538^*^
Median (1st”3rd. quartiles)	9.38 (7.08”13.79)	12.71 (10.68”13.62)	13.58 (7.88”20.21)	
Sex				
Female	26 (33.3%)	2 (50.0%)	5 (45.5%)	0.5767†
Male	52 (66.7%)	2 (50.0%)	6 (54.5%)	
Hb at admission				
N	75	4	11	0.4738^*^
Median (1st”3rd. quartiles)	11.10 (9.30”12.50)	12.05 (11.03”12.90)	11.30 (8.15”11.80)	
Hb at last consultation				
N	73	4	11	0.8126^*^
Median (1st”3rd. quartiles)	12.90 (11.5”14.2)	13.25 (12.62”13.82)	13.40 (12.35”13.95)	
R at admission				
N	68	4	10	0.4094^*^
Median (1st”3rd. quartiles)	1.16 (1.03”1.29)	1.33 (1.22”1.61)	1.13 (1.08”1.32)	
R at last consultation				
N	68	4	11	0.0026^*^#
Median (1st”3rd. quartiles)	1.10 (1.03”1.20)	1.04 (1.02”1.05)	0.99 (0.96”0.99)	
Difference between: 1 and 3‡				
INR at admission				
N	71	4	11	0.6860^*^
Median (1st”3rd. quartiles)	1.26 (1.13”1.37)	1.23 (1.15”1.32)	1.31 (1.18”1.42)	
Difference between: 1 and 3‡				
INR at last consultation				
N	72	4	11	0.0038^*^,‡
Median (1st”3rd. quartiles)	1.31 (1.19”1.48)	1.31 (1.25”1.38)	1.12 (1.06”1.25)	
Platelets at admission				
N	73	4	11	0.8230^*^
Median (1st”3rd. quartiles)	107,000 (59,000”159,000)	72,000 (64,250”106,000)	91,000 (58,000”169,000)	
Platelets at last consultation				
N	73	4	11	0.0001^*,‡^
Median (1st”3rd. quartiles)	69,000 (46,000”105,000)	58,000 (44,500”74,000)	324,000 (281,500”351,500)	
Difference between: 1 and 3; 2 and 3^‡^				
Splenic index at admission				
N	56	4	7	0.7214^*^
Median (1st”3rd. quartiles)	1.38 (1.21”1.57)	1.39 (1.36”1.42)	1.48 (1.31”1.60)	

^*^Based on Kruskal-Wallis or Mann-Whitney tests

^†^Based on Fisher’s exact test

^‡^Based on Dunn’s test.

N: number of patients; SD: standard deviation; Hb: hemoglobin; INR: international normalized ratio; R: thromboplastin time ratio.

When comparing groups 1, 2, and 3, it is observed that at admission, there is no significant difference in the values of R, INR, and platelet count. However, at the last follow-up, significant differences were noted. For R and INR, the difference is established between groups 1 and 3. For platelet count, a difference is observed between groups 1 and 3. Therefore, it is noted that at the last follow-up, there was a significant reduction in R and INR, and a significant increase in platelet count in group 3 compared to group 1.

Table 2 presents the results of coagulation-related tests in groups 1 and 3 at the time of admission and at the last follow-up. The primary comparisons were focused on groups 1 and 3, as group 2 included a limited number of patients.

**Table 2:** Comparison of thromboplastin time ratio, international normalized ratio, and platelet values betweengroups 1 and 3 and between time points (admission and last consultation).

Variable	Group 1	Group 3	p-value^*^
R at admission^*^			
N	63	10	0.8788
Median (1st”3rd. quartiles)	1.16 (1.03”1.29)	1.13 (1.08”1.32)	
R at last consultation			
N	63	10	
Median (1st”3rd. quartiles) p-value (group-time interaction): 0.0187†	1.10 (1.03”1.20)	0.99 (0.96”1.00)	0.0057
INR at admission^*^			
N	68	11	0.4921
Median (1st”3rd. quartiles)	1.27 (1.13”1.39)	1.31 (1.18”1.42)	
INR at last consultation			
N	68	11	0.0014
Median (1st”3rd. quartiles)	1.31 (1.18”1.48)	1.12 (1.06”1.25)	
p-value (group-time interaction): 0.0013^†^			
Platelets at admission^*^			
N	70	11	0.8362
Median (1st”3rd. quartiles)	103,000 (58,250”158,250)	91,000 (58,000”169,000)	
Platelets at last consultation			
N	70	11	0.0001
Median (1st”3rd. quartiles)	66,500 (45,250”99,250)	324,000 (281,500”351,500)	
p-value (group-time interaction)<0.0001†			

^*^ p-value (Mann-Whitney) applied to the variables only at admission

^†^ Results of the ANOVA for repeated measures with rank transformation.

N: number of patients; SD: standard deviation; INR: international normalized ratio; R: thromboplastin time ratio.

When comparing groups 1 and 3, a statistically significant difference is observed when analyzing R, INR, and platelets separately at two time points: upon admission and at the last consultation.

In the analysis of R, group 1 (G1) showed an average of 1.16 at admission and 1.10 at the last consultation, while group 3 (G3) had an average of 1.13 at admission and 0.99 at the last consultation. When applying the Mann-Whitney test, no significant difference was found between groups 1 and 3 at admission (p=0.8788). However, the ANOVA test revealed a group-time interaction p-value of 0.0187. Since the group-time interaction was significant, further breakdowns of the factors involved were performed. The first breakdown was the comparison between groups 1 and 3 at the last consultation, which resulted in a significant difference (p=0.0057). The second breakdown involved comparing the time points within each group (difference between admission and last consultation within the same group). In group 1 (G1), there was no significant difference (p time G1=0.1806) between the R values at admission and the last consultation. However, in group 3 (G3), a significant difference was observed (p time G3=0.022) between the R values at admission and the last consultation. Therefore, when analyzing R, it can be concluded that there was a significant reduction in the R value at the last consultation in G3 compared to G1. In the separate analysis of each group, it was observed that in group 1 (G1), there was no significant change between admission and the last consultation, whereas in group 3 (G3), there was a significant reduction between admission and the last consultation.

In the analysis of INR, group 1 (G1) showed an average of 1.27 at admission and 1.31 at the last consultation, while group 3 (G3) had an average of 1.31 at admission and 1.12 at the last consultation. When the Mann-Whitney test was applied, no significant difference was found between groups 1 and 3 at admission (p=0.4921). However, when the ANOVA test was performed, a group-time interaction p-value of 0.0013 was observed. Since the group-time interaction was significant, further breakdowns of the factors involved were conducted. The first breakdown was the comparison between groups 1 and 3 at the last consultation, which revealed a significant difference (p=0.0014). The second breakdown involved comparing the time points within each group (the difference between admission and last consultation within the same group). In group 1 (G1), there was a significant difference (p time G1=0.0422) between the INR at admission and at the last consultation, and in group 3 (G3), a significant difference was also observed (p time G3=0.0305) between the INR at admission and at the last consultation. Therefore, when analyzing INR, it can be concluded that there was a significant reduction in the INR value at the last consultation in G3 compared to G1.In the separate analysis of each group, it was observed that in group 1 (G1), there was a significant increase between admission and the last consultation, whereas in group 3 (G3), there was a significant reduction between admission and the last consultation.

In the analysis of platelets, group 1 (G1) showed an average of 103,000/mm^3^ at admission and 66,500/mm^3^ at the last consultation, while group 3 (G3) had an average of 91,000/mm^3^ at admission and 324,000/mm^3^ at the last consultation. When the Mann-Whitney test was applied, no significant difference was observed between groups 1 and 3 at admission (p=0.8362). However, when the ANOVA test was performed, a group-time interaction p-value of <0.0001 was observed. Since the group- time interaction was significant, further breakdowns of the factors involved were conducted. The first breakdown was the comparison between groups 1 and 3 at the last consultation, which resulted in a significant difference (p<0.0001). The second breakdown involved comparing the time points within each group (difference between admission and last consultation within the same group). In group 1 (G1), a significant difference was observed (p time G1<0.0001) between the platelet values at admission and the last consultation, and in group 3 (G3), a significant difference was also observed (p time G3=0.0011) between the platelet values at admission and the last consultation. Therefore, when analyzing platelets, it can be concluded that there was a significant increase in platelet count at the last consultation in G3 compared to G1. In the separate analysis of each group, it was observed that in G1 there was a significant reduction between admission and the last consultation, while in G3 there was a significant increase between admission and the last consultation. This reinforces the idea that marked splenomegaly reduces platelet count, and splenectomy may be responsible for the increase.

Table 3 presents the mean and median values of platelet count in group 3 before and after the splenectomy surgery.

**Table 3:** Comparison of platelet values in group 3 between time points (before and after surgery).

Variable	Group 3 (n=10)	p-value (Wilcoxon)
Platelets before surgery Median (1st”3rd. quartiles)	37,000 (28,750”70,250)	
Platelets after surgery Median (1st”3rd. quartiles)	273,000 (100,250”563,750)	
Difference Median (1st”3rd. quartiles)	206,500 (33,250”529,500)	0.0020

When group 3 was individually analyzed using the Wilcoxon test for related samples, a statistically significant difference in platelet count was observed (p=0.002) when comparing before and after the splenectomy.

Table 4 presents the spleen index values at admission and at the last consultation for patients in group 1.

**Table 4:** Comparison of splenic index between time points in group 1.

Variable	Group 1 (n=51)	p-value (Wilcoxon)
Splenic index at admission Median (1st”3rd. quartiles)	1.39 (1.22”1.59)	
Last splenic index Median (1st”3rd. quartiles)	1.57 (1.37”1.77)	
Difference Median (1st”3rd. quartiles)	0.19 (0.05”0.34)	<0.0001

When group 1 was individually analyzed using the Wilcoxon test for related samples, a statistically significant difference in the spleen index was observed (p<.0001) when comparing admission and the last consultation.

## DISCUSSION

The results presented in this study show that pediatric patients diagnosed with EHPVO exhibit a high percentage of thrombocytopenia and prolonged INR from admission. These findings are likely due to the consumption of coagulation factors and the rapid and premature destruction of platelets by the spleen.

It is important to emphasize the high frequency of prolonged INR in patients with EHPVO and the possible pathophysiology underlying this result. Few studies have explored this topic. One of them reported a prevalence of 35.8% of prolonged INR in a cohort of 45 patients with EHPVO.^7^ In the cohort of the present study (n=93), a higher percentage was observed (52.3%). This information is relevant because, in the presence of a patient with upper gastrointestinal bleeding due to esophageal varices and prolonged INR, some professionals may consider the possibility of chronic liver disease and focus the investigation in that direction, including performing a liver biopsy — a procedure that is not necessary in this condition to establish the diagnosis.

Regarding the pathophysiology of coagulation disorders, studies report that the abnormalities are likely due to persistent activation of endotoxins. Additionally, they state that hypersplenism does not significantly influence the coagulation mechanisms.^18,19,20^ Another mechanism proposed in the literature to explain the coagulation factor disturbances in patients with EHPVO is hepatic hypoperfusion, as the liver is an essential organ for the synthesis of these coagulation factors. A study demonstrated that after performing a mesoportocaval shunt (Rex shunt) in patients with EHPVO, there was a significant increase in factors V and VII, as well as a significant shortening of prothrombin time.^22^

None of the studies mentioned above analyzed the results in patients who underwent splenectomy to determine whether the coagulation changes could be explained by the enlargement of the spleen.

The present study demonstrated that there are significant changes in platelet count, INR values, and R values in pediatric patients diagnosed with EHPVO who underwent splenectomy compared to those who did not undergo the surgery. This result suggests that in the enlarged spleen of these patients, processes may occur that lead to a decrease in coagulation factors, in addition to platelets. This alteration is supported by a study, in which a statistically significant decrease in the activity of factors II, V, VII, and VIII was observed in the group of patients with a diagnosis of portal vein obstruction without liver parenchymal involvement, when compared to the group of healthy patients.^23^

Thus, the present research indicates that the likely mechanism for the prolonged INR and R is the consumption of coagulation factors by the enlarged spleen, as INR and R return to normal in patients who undergo splenectomy. This hypothesis is original and has not yet been proposed in the medical literature, thereby contributing to the understanding of the pathogenesis of coagulation and platelet abnormalities in these patients. So, how can we challenge the hypothesis of hypoperfusion as suggested by Mack et al.^22^ According to the study, the evaluation of the increase in coagulation factors took place 1 year after the surgical procedure, a time when the spleen likely decreased in size, and this fact could explain the increase in these factors. Another argument against the hypothesis that hepatic hypoperfusion would explain the reduction in coagulation factors, and that normalization of this flow through Rex shunt surgery would restore coagulation factors to normal levels, is the fact that none of the patients in group 3 of the present study, who underwent splenectomy, had Rex shunt surgery, yet their coagulation was restored to normal levels.

The splenic index increased in the last evaluation of group 1 and showed a negative correlation with platelet count. These results suggest that spleen enlargement is a natural progression of the disease and that the decrease in platelet count is a consequence of this. The appearance of a cavernoma shortly after thrombosis is an attempt to restore blood flow to the affected area, but this is not effectively achieved in most cases. Thus, as portal hypertension develops, the spleen continues to enlarge; furthermore, the treatment of bleeding varices results in increased hypertension at other sites, including the spleen.

The limitations of this study include the need to add, in the hematological evaluation, the dosages of coagulation factors involved in the extrinsic and intrinsic pathways, both before and after surgical procedures. Additionally, the retrospective and single-center design of the study limits the sample size, particularly the number of patients in group 3. Therefore, it is necessary to expand the series to confirm or refute the findings of the present research.

In conclusion, based on the results presented, it can be concluded In the INR analysis that there was a high percentage of anemia, thrombocytopenia, and prolonged INR at admission. It can also be stated that splenectomy resulted in an increase in platelet count and a reduction in R and INR. Therefore, the alteration in INR and R at admission is not due to the consumption of coagulation factors in the cavernous region, but rather, most likely in the spleen.

## Data Availability

The database that originated the article is available with the corresponding author.

## References

[1] Sarin SK, Sollano JD, Chawla YK, Amarapurkar D, Hamid S, Hashizume M (2006). Consensus on extra-hepatic portal vein obstruction.. Liver Int.

[2] Warren WD, Millikan WJ, Smith RB, Rypins EB, Henderson JM, Salam AA (1980). Noncirrhotic portal vein thrombosis. Physiology before and after shunts.. Ann Surg.

[3] De Gaetano AM, Lafortune M, Patriquin H, De Franco A, Aubin B, Paradis K (1995). Cavernous transformation of the portal vein: patterns of intrahepatic and splanchnic collateral circulation detected with Doppler sonography. AJR Am J Roentgenol.

[4] Sarin SK, Agarwal SR (2002). Extrahepatic portal vein obstruction. Semin Liver Dis.

[5] Abdel-Ghaffar TY, Zakaria H, El Naghi S, Elsayed SM, Haseeb A, Sobhy GA (2023). Extra-hepatic portal vein thrombosis in children: single center experience. Clin Exper Hepatol.

[6] Sooraj K, Shivani F, Khan MH, Kumar RR, Bai S, Hussaini H (2022). Frequency of causes of portal hypertension in children.. Cureus.

[7] Yayla EN, Siri S, Kaya NG, G?rkan OE, S‘zen H, ¥zen IO (2023). Portal hypertension in children: a tertiary center experience in Turkey. Pediatr Gastroenterol Hepatol Nutr.

[8] Zwiebel WJ (1995). Sonographic diagnosis of hepatic vascular disorders. Semin Ultrasound CT MR.

[9] Zhang J, Li L (2022). Rex shunt for extra-hepatic portal venous obstruction in children.. Children (Basel).

[10] Vadlapudi SS, Jagadisan B, Ananthkrishnan R, Narayanaswamy S (2020). Splenic stiffness and platelet count to predict varices needing treatment in pediatric extrahepatic portal vein obstruction. Indian J Gastroenterol.

[11] Yokoyama S, Ishizu Y, Honda T, Imai N, Ito T, Yamamoto K (2022). Pipeline esophagogastric varices secondary to extrahepatic portal vein obstruction treated endoscopically with the assistance of transileocolic obliteration. Intern Med.

[12] Duché M, Ducot B, Ackermann O, Guérin F, Jacquemin E, Bernard O (2017). Portal hypertension in children: High-risk varices, primary prophylaxis and consequences of bleeding. J Hepatol.

[13] Éboli LP, Matias MC, Cordeiro RN, Araújo GM, Souza EA, Lima DL (2018). Avaliação da média de ressangramento em pacientes pediátricos com trombose da veia porta que fizeram uso de profilaxia endosc—pica e/ou medicamentosa. Rev Med.

[14] Giouleme O, Theocharidou E (2013). Management of portal hypertension in children with portal vein thrombosis. J Pediatr Gastroenterol Nutr.

[15] Kim SK, Lee KA, Sauk S, Korenblat K (2017). Comparison of transjugular intrahepatic portosystemic shunt with covered stent and balloon- occluded retrograde transvenous obliteration in managing isolated gastric varices. Korean J Radiol.

[16] Alvarez F, Bernard O, Brunelle F, Hadchouel P, Odi?vre M, Alagille D (1983). Portal obstruction in children. I. Clinical investigation and hemorrhage risk.. J Pediatr.

[17] Prasad CV, Kaur U, Marwaha N, Ghosh K, Chawla YK, Dilawári JB (1990). Hemostatic alterations in non-cirrhotic portal fibrosis, extrahepatic portal venous obstruction and Budd-Chiari syndrome. Indian J Gastroenterol.

[18] Sheth SG, Deo AM, Bichile SK, Amarapurkar DN, Chopra KB, Mehta PJ (1996). Coagulation abnormalities in non-cirrhotic portal fibrosis and extra hepatic portal vein obstruction. J Assoc Physic India.

[19] Robson SC, Kahn D, Kruskal J, Bird AR, Kirsch RE (1993). Disordered hemostasis in extrahepatic portal hypertension. Hepatology.

[20] Bajaj JS, Bhattacharjee J, Sarin SK (2001). Coagulation profile and platelet function in patients with extrahepatic portal vein obstruction and non-cirrhotic portal fibrosis. J Gastroenterol Hepatol.

[21] Konuş OL, Ozdemir A, Akkaya A, Erbaş G, Celik H, Işik S (1998). Normal liver, spleen, and kidney dimensions in neonates, infants, and children: evaluation with sonography. AJR Am J Roentgenol.

[22] Mack CL, Superina RA (2003). Whitington PF Surgical restoration of portal flow corrects procoagulant and anticoagulant deficiencies associated with extrahepatic portal vien thrombosis.. J Pediatr.

[23] Beattie W, Magnusson M, Hardikar W, Monagle P, Ignjatovic V (2017). Characterization of the coagulation profile in children with liver disease and extrahepatic portal vein obstruction or shunt. Pediatr Hematol Oncol.

